# Morphology and Performance of Polymer Solar Cell Characterized by DPD Simulation and Graph Theory

**DOI:** 10.1038/srep16854

**Published:** 2015-11-19

**Authors:** Chunmiao Du, Yujin Ji, Junwei Xue, Tingjun Hou, Jianxin Tang, Shuit-Tong Lee, Youyong Li

**Affiliations:** 1 Institute of Functional Nano and Soft Materials (FUNSOM), Soochow University, Suzhou, Jiangsu 215123, P.R. China

## Abstract

The morphology of active layers in the bulk heterojunction (BHJ) solar cells is critical to the performance of organic photovoltaics (OPV). Currently, there is limited information for the morphology from transmission electron microscopy (TEM) techniques. Meanwhile, there are limited approaches to predict the morphology /efficiency of OPV. Here we use Dissipative Particle Dynamics (DPD) to determine 3D morphology of BHJ solar cells and show DPD to be an efficient approach to predict the 3D morphology. Based on the 3D morphology, we estimate the performance indicator of BHJ solar cells by using graph theory. Specifically, we study poly (3-hexylthiophene)/[6, 6]-phenyl-C_61_butyric acid methyl ester (P3HT/PCBM) BHJ solar cells. We find that, when the volume fraction of PCBM is in the region 0.4 ∼ 0.5, P3HT/PCBM will show bi-continuous morphology and optimum performance, consistent with experimental results. Further, the optimum temperature (413 K) for the morphology and performance of P3HT/PCBM is in accord with annealing results. We find that solvent additive plays a critical role in the desolvation process of P3HT/PCBM BHJ solar cell. Our approach provides a direct method to predict dynamic 3D morphology and performance indicator for BHJ solar cells.

Organic photovoltaics (OPV) based on polymer/fullerene mixtures have attracted wide attention for decades due to their low-cost and flexibility^1^,^2^,^3^. Most OPV consist of a single bulk-heterojunction (BHJ) active layer, in which the electron donor (conjugated polymer) and electron acceptor (fullerene) are deposited from a common solvent. To achieve efficient exciton dissociation and charge transport, an interpenetrating network of electron-donor (D) and -acceptor (A) domains on a length scale of the exciton diffusion length within the active layer is required and introduced during the deposition/drying process or post-production treatment. Thus, besides the chemical composition or molecular architecture, the morphology of the active layer on different length scales also significantly contributes to the overall performance of polymer solar cells (PSCs)^3^,^4^,^5^,^6^. Consequently, the influence of typical control parameters, such as blending ratio, chemical structure, solvent, concentration in solution and post-production treatments, on the morphology of polymer-based BHJ systems have been investigated intensively by the recent experimental techniques like electron tomography and advanced scattering techniques^4^. Currently, the 3D morphology characteristics have been realized by some experimental scattering techniques including neutron or X-ray scattering, ellipsometry, dynamic secondary ion mass spectrometry or transmission electron microscopy in tomography mode^4^,^7^,^8^. Dynamic Monte Carlo^9^,^10^,^11^,^12^,^13^,^14^,^15^,^16^ or graph theory^17^ have been used to predict the efficiency of BHJ solar cells based on randomly generated morphology^17^, Ising model^12^,^13^, or cellular automata model^11^. In addition, the coarse-grained molecular simulation studies of bulk heterojunctions were also reported^18^,^19^,^20^,^21^,^22^. Here we realize a novel DPD simulation method to characterize the 3D dynamic morphology of OPV system, which is better than the static limited scanning probe methodologies.

Here we first perform atomistic molecular dynamics simulation to obtain interaction parameters for the components of the active layer of OPV. Then we perform Dissipative Particle Dynamics (DPD)^23^ to obtain simulated equilibrated morphology of the active layer of OPV. Based on the predicted 3D morphology, we estimate the performance indicator by using graph theory^17^. And this performance indicator is defined in the second part of the Methods section: Characterization of morphology based on morphology descriptors. We prove that DPD is an efficient approach to predict 3D morphology of BHJ solar cells. DPD is an NVT method to simulate a Hamiltonian system in the canonical ensemble. However, DPD preserves hydrodynamics, which is important in simulated solvent annealing defects in ordered mesophases^24^. And it is able to consider shearing directly. Thus DPD has an intrinsic advantage over other methods such as dynamic density functional theory or Monte Carlo methods, in following the evolution of a system towards an ordered thermodynamic equilibrium state. The internal degrees of freedom of particles are integrated out and replaced by simplified pairwise dissipative and random forces, so as to conserve momentum locally and ensure correct hydrodynamic behavior. Compared with usual molecular dynamics (MD) simulations, DPD uses soft potential to describe inter-molecular interactions. The soft potential allows for a much larger time step than is commonly used in usual MD simulations. Our DPD simulation results indicate that DPD is an efficient approach to determine 3D morphology of BHJ solar cells. Our results provide dynamic 3D morphology and elucidate the critical factors affecting the desolvation process and equilibrium morphology for BHJ solar cells.

Based on the morphology of BHJ solar cells from DPD simulations, we estimate the performance indicator by using graph theory^17^. In 2012, Wodo *et al.*^17^ presented a graph-based framework to efficiently compute a broad suite of physically meaningful, morphology descriptors. With these morphology descriptors, Wodo *et al.*^17^ are able to characterize and quantify the morphologies. Here we use similar approach to characterize the 3D morphologies obtained from our DPD simulations. We find that, when the volume fraction of PCBM is in the region 0.4 ∼ 0.6, PCBM/P3HT will show bi-continuous morphology and optimum performance indicator, which is consistent with experimental results. Our results indicate that there is an optimum temperature (413K) for P3HT/PCBM’s morphology and performance indicator, which is consistent with annealing results. And we obtain domain size consistent with TEM analysis results. We find that solvent additive plays a critical role in the desolvation process of PCBM/P3HT BHJ solar cell, which improves the phase separation and the equilibrium morphology of the active layer.

## Results and Discussion

### The dependence of the morphology and performance indicator on the blend ratio

It is well known that the corresponding nanoscale structure of P3HT/PCBM strongly depends on the ratio of the components involved. For the system P3HT: PCBM, an optimum blending ratio around 50 wt % was reported from several groups^4^,^25^,^26^,^27^.

**Figure 1:**
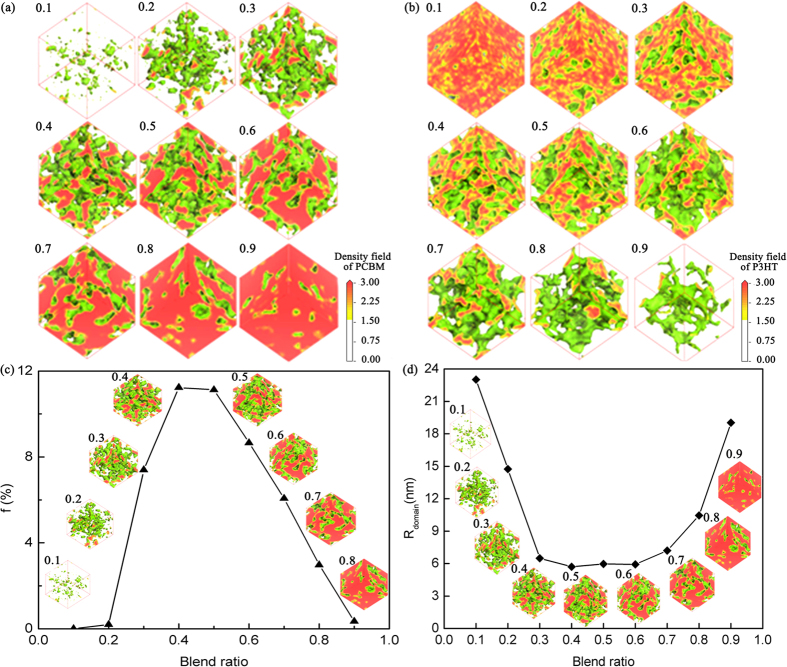
T = 298K, (**a**) the density field of PCBM and (**b**) P3HT with various amount of PCBM(from 0.1–0.9). (**c**) Performance indicator f and (**d**) domain size of the P3HT/PCBM system evaluated from DPD simulation as a function of blend ratio. Blend ratio is represented by the volume percentage of PCBM. Domain size is defined as the volume divided by the interface area.

Here we study the P3HT/PCBM with the volume percentage of PCBM varying from 0.1, 0.2, 0.3, 0.4, 0.5, 0.6, 0.7, 0.8, to 0.9 at room temperature. Figure 1(a,b) show the density fields for PCBM and P3HT with different blend ratio. We can see that only for PCBM volume percentage = 0.4, 0.5, or 0.6, the morphology is bi-continuous nanoscale morphology. For PCBM volume percentage = 0.7 – 0.9, the density field of PCBM doesn’t form continuous percolating morphology, but isolated channel of P3HT density field. For PCBM volume percentage = 0.1–0.3, the density field of P3HT doesn’t form continuous percolating morphology, but isolated channel of PCBM density field or not even grain-like morphology.

With morphologies obtained as shown in Fig. 1, we use graph theory to evaluate the performance indicator and the results are shown in Fig. 1(c). It shows that when the PCBM volume percentage is around 0.4 ∼ 0.5, the performance indicator is optimal. The domain size as a function of blend ratio is shown in Fig. 1(d). We can see that, when the PCBM volume percentage is around 0.4 ∼ 0.6, the domain size is around 6 nm, which is a favorable size for the performance indicator of the active layer. The optimum domain size favorable for performance indicator is around 6 nm.

Thus high performance indicator morphology is formed for volume fraction of PCBM in the region of 0.4 ∼ 0.5, which corresponds to 0.52 ∼ 0.61 weight percentage. Experimental studies of the impact of blending composition on device performance suggested that an optimum PCBM blending ratio was around 40 ∼ 60 wt% for the P3HT:PCBM system^26^,^28^,^29^,^30^ and 80 wt% for the MDMO-PPV:PCBM system^27^. In general, the aim of optimum blending ratio in BHJ systems is to obtain a bi-continuous, percolating network for the charge carrier transport. In blends of lower or higher loading of PCBM, absence of the PCBM or P3HT percolating network throughout the thickness of the photoactive layer would lead to unbalanced charge transport and bad device performance.

### The dependence of the morphology and performance indicator on temperature

Heating has been proved to be one of the most effective methods to control and enhance the phase separation in the P3HT/PC_61_BM system. Upon changing the heating temperature, the extent of phase separation evolves and finally forms phase-separated network-like morphology. Thus the temperature is an important parameter for morphology control^31^,^32^.

Here we perform DPD simulation of P3HT/PCBM system to investigate the effect of the temperature. To give an estimate of the values of the reduced temperatures in terms of physical temperatures, we have mapped the reduced temperatures, T*, onto physical temperatures, T, according to the linear relation below. We obtain the interaction parameters of DPD particles at 298 K and the reduced physical temperature in DPD: T* = 1 corresponds to T = 298 K. To simplify the relationship between T and T*, we use the following relationship between T and T*: T = a*T, where a = 298, T and T* represent the physical temperature and reduced temperature, respectively^33^,^34^.

**Figure 2:**
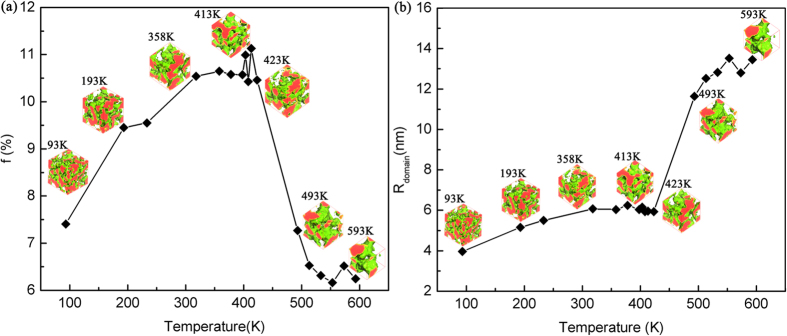
(**a**) Performance indicator f and (**b**) domain size for 1:1 vol P3HT/PCBM blend evaluated from DPD simulation as a function of temperature. Domain size is defined as the volume divided by the interface area. The optimum domain size favorable for performance indicator is around 6 nm.

The density fields of PCBM (for 1:1 vol P3HT/PCBM blend) equilibrated at different temperatures 93K ∼ 593K in our DPD simulations are shown in Fig. 2. The density fields of PCBM are colored with red. The green curved surface, i.e. isosurface of density field at *θ*_PCBM_ = 1.50, stands for the interface between P3HT and PCBM.

Figure 2(b) shows that the domain size depends on the temperature. The domain size is defined as the volume divided by the interface area. Here we can see that, the phase separation of PCBM becomes stronger with enhanced temperature. The domain size increases with increasing temperature. After we obtain the morphology, we use graph theory to evaluate the performance indicator and the results are shown in Fig. 2(a). When the temperature is around 413 K, the domain size is around 6 nm as shown in Fig. 2(b) and the performance indicator is optimum. These are consistent with experimental results^31^,^35^,^36^, showing that annealing is an efficient way to improve the morphology/performance indicator of the P3HT/PCBM OPV and 140 °C ∼ 150 °C is a favorable annealing temperature. In general, increase in temperature is favorable for enhancing the phase separation and increasing the domain size of P3HT/PCBM blend. When the domain size is around 6 nm, it shows optimum performance indicator. Quantitatively, the domain size of the morphologies calculated with our DPD model tends to saturates at nearly 13 nm around 550K in Fig. 2(b).

### Morphology/performance indicator during solvent’s evaporation with and without additive

The spin-casting technique is generally used for the fabrication of BHJ solar cells. During solvent evaporation, phase separation of components will occur in the spin-coating process^6^. Thus, the initial morphology of spin-casting from diluted solution is the result of a kinetically frozen phase separation or crystallization and far from thermodynamically equilibrium morphology^29^.

**Figure 3:**
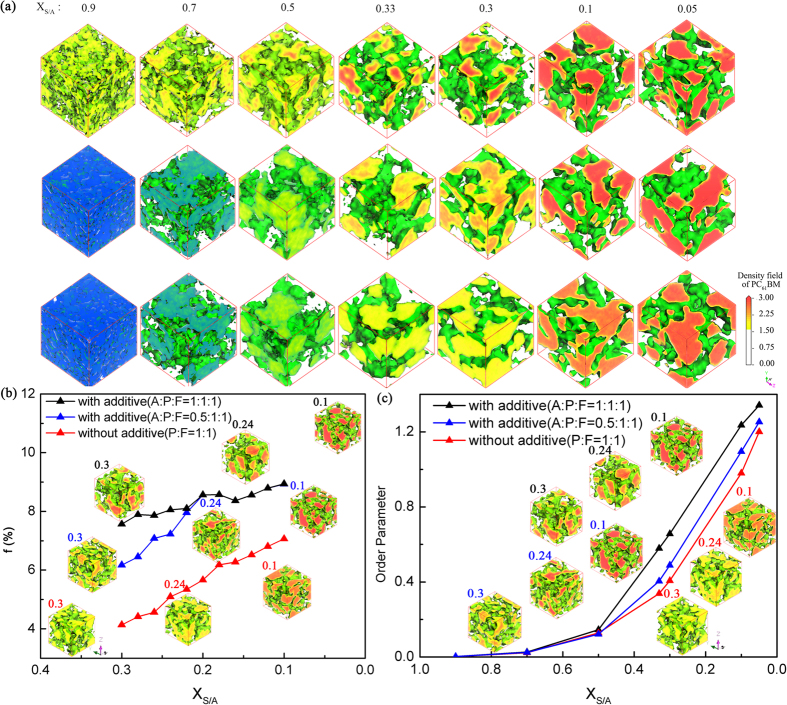
(**a**) Predicted morphology of PCBM during the solvent evaporation with various amounts of additives (the first and the second row) or without additive (the third row). (**b**) Performance indicator f for 1:1 vol P3HT/PCBM blend evaluated from DPD simulation during desolvation process. (**c**) The order parameters as a function of the solvent percentage. (A, P, F stand for additive, P3HT, PCBM respectively).

We investigate the simulated equilibrium morphology of P3HT/PCBM during the spin-casting process or solvent evaporation by using DPD simulations. The simulated equilibrium morphologies of the ternary system without 1, 8-octanedithiol as additive after 50000 simulation steps of DPD are shown in the third row of Fig. 3(a). The percentage X_S/A_ indicates the percentage of the chlorobenzene (CB) solvent plus the additive in the P3HT/PCBM system. From left to the right, X_S/A_ decreases from 0.9 to 0.05 as the solvent evaporates. For all systems, the ratio between P3HT and PCBM is 1:1, which is an optimum ratio for the performance indicator. The ratio of (Additive: P3HT: PCBM) (A: P: F) is 1:1:1 for the first row; 0.5:1:1 for the second row; 0:1:1 for the third row. It indicates that additive helps to improve the phase separation between P3HT and PCBM.

Figure 3(a) shows that phase separation is enhanced during the desolvation process (from the left to the right). This is confirmed by the plot of order parameters of PCBM particle in Fig. 3(c).

We characterize the phase separation by the order parameters for each component. The order parameter, P_i_, is defined as the volume average of the difference between the local density squared and the overall density squared, the order parameter is defined as in the following equation:


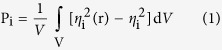


Where *η*_*i*_ is a dimensionless density (volume fraction) for species *i*, *η*_*i*_*(r)* = *νρ*_*i*_*(r)* where *ρ*_*i*_*(r)* represents the practical local physical density for species *i*, ν is a scale factor between the local density *η*_*i*_*(r)* in DPD and *ρ*_*i*_*(r)*. We use a simple script to calculate the Order parameter of F particle in the system. The script can be found in Supplementary Information S4. Order parameters with large values indicate strong phase segregation.

From the Fig. 3(c), we can see that the order parameter change obviously with the addition of solvents and additives. When the fraction of solvents and additives decreases from 50% to none, the order parameter of P3HT: PCBM with additive (A: P: F = 1:1:1) and (A: P: F = 0.5:1:1) is higher than the system with none solvents and additives, which means that the function of solvents and additives is to accelerate the phase separation of morphology of liquid system. This agrees well with experiments^37^. Figure 3(c) indicates that additive helps to improve the phase separation between P3HT and PCBM.

When the solvent concentration is 0.9, the PCBM molecules distribute homogeneously in 3-dimensions and there is little phase separation. The sharply increase of order parameter in Fig. 3(c) indicates the aggregation of PCBM and the phase separation when the solvent/additive amount is reduced to 0.5. It indicates that the different amount of additive helps to improve the phase separation of the system. The higher amount of the additive introduces bigger phase separation.

Meanwhile, we use graph theory to evaluate the performance indicator of the morphologies obtained in Fig. 3(c). The results are shown in Fig. 3(b). We can see that, during the desolvation process, there is less solvent and the phase separation becomes stronger and the performance indicator increases. When there are more additives, the phase separation is enhanced and the performance indicator increases as well.

The simulated equilibrium morphologies with various amount of solvent represent the dynamical process^38^ during solvent evaporation for polymer/fullerene/solvent ternary system. Processing additive^39^,^40^,^41^(1, 8-octanedithiol) on morphology^42^ has been proved effective during film fabrication. With additive, the phase separation happens earlier during desolvation process of P3HT/PCBM^39^,^43^. Our results show that the additive helps to increase the phase separation and the performance indicator of P3HT/PCBM active layer^44^,^26^, revealing the underlining mechanism of the effect of the additives.

During film processing, the solvent evaporates earlier than the additive. The function of the host solvent CB is to get a uniform distributed blend at a high spin-cast speed during film fabrication. Once the host solvent extract from the system completely (X_S/A_ = 0.33, 0.3, 0.1, 0.05), the selective solubility of additive towards one of components in the blend, PCBM, impels the nanoscale aggregation of another component (P3HT).

**Figure 4:**
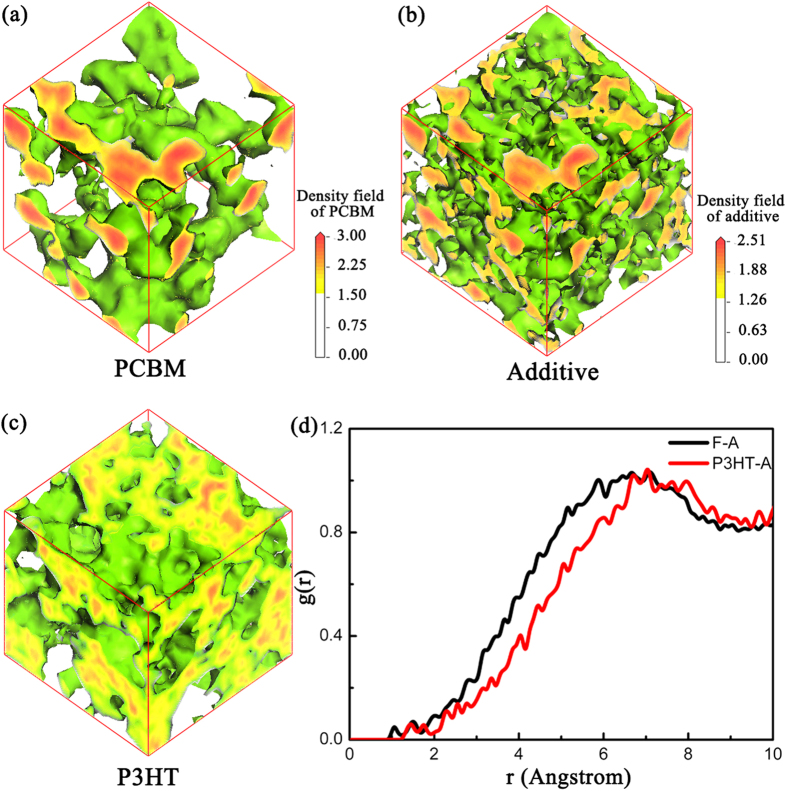
The density field distribution of (**a**) PCBM, (**b**) additive and (**c**) P3HT when X_S/A_ = 0.33. (**d**) The RDF (radial distribution function) of the moiety F(PCBM)-A(additive) and the moiety P3HT-A(additive) when X_S/A_ = 0.33; g(r)_F-A shows lower value than g(r)_P3HT-A, which indicates that the additive distributes mainly in PCBM region. The selective solubility of additive towards PCBM enhances the phase separation and the device performance.

Figure 4 provides the density field distribution of P3HT (Fig. 4(a)), additive (Fig. 4(b)), and PCBM (Fig. 4(c)) when X_S/A_ = 0.33(X_S_ = 0.00; X_A_ = 0.33). It shows that the additive distributes mainly in PCBM region. Figure 4(d) provides the quantitative analysis of RDF (Radial Distribution Function) distribution of g(r)_PCBM-Additive and g(r)_P3HT-Additive^39^,^45^. Obviously, the peak of g(r)_PCBM-Additive is smaller than that of g(r)_P3HT-Additive. It indicates that the additive distributes closer to PCBM instead of P3HT. And the additive is distributed in the PCBM region rather than P3HT region. The selective solubility of additive in PCBM enhances the phase separation and the device performance^37^.

The morphology of active layers in the BHJ solar cells is critical to the performance of OPV. Although, the 3D morphology characteristic have been realized by some experimental scattering techniques including neutron or X-ray scattering, ellipsometry, dynamic secondary ion mass spectrometry or transmission electron microscopy in tomography mode^4^,^7^,^8^. Currently, the approach to predict the morphology and performance indicator of OPV is still limited. Here we determine 3D morphology of BHJ solar cells by using Dissipative Particle Dynamics (DPD), and show DPD is an efficient approach to predict the 3D morphology of BHJ solar cells. Based on the predicted 3D morphology, we estimate the performance indicator of BHJ solar cells by using graph theory.

Our simulation results show that the extent of phase separation between polymer and fullerene becomes larger during solvent evaporation. The additive induces the aggregation of one of the components and improves the phase separation, especially when the host solvent is almost evaporated totally. Increased device temperature results in stronger phase separation and larger domain size of PCBM. We also study the impact of P3HT/PCBM blending ratio on the equilibrated morphology. We obtain the same optimum blend ratio as experimental results, which is important for P3HT and PCBM to form bi-continuous network. Importantly, our results provide dynamic 3D morphology and reveal the critical factors affecting the desolvation process and equilibrium morphology for BHJ solar cells.

P3HT or PCBM always shows certain crystallinity in the P3HT/PCBM blend, which affects its performance indicator directly. For example, Mayer *et al.*^46^ showed that the ideal blend ratio of a particular system is often due to details of the crystallinity of the polymer.

We calculate “Spatial Orientation Correlation Function” (SOCF) to characterize the crystallinity of the system (See Supplementary Figure S5). As indicated by Lee *et al.*^18^, a typical liquid crystal has an order parameter in the range 0.3 to 0.1. Our results show that SOCF is below 0.3, which indicates the low crystallinity in our simulation box. It is caused by the small size of our simulation box. In addition, we don’t consider the effect of HOMO/LUMO energy levels. These simplifications lead to the overestimated performance indicator from our approach.

For OPV systems such as PTB7:PCBM^47^ don’t have high crystallinity and the nanostructure formation is driven more from interaction parameters. It will be of interest to study PTB7:PCBM system with our approach.

Our work here provides an approach to predict the morphology/performance indicator of the photoactive layer in BHJ PSCs based on the chemical structures of the components. Characterization and optimization of the different device parameters, such as temperature, blend ratio, solvent effect, and chemical structure, provide important guidance to improve the performance of OPV.

## Methods

### DPD simulations to obtain morphology of BHJ solar cells

Dissipative particle dynamics (DPD) was initially proposed by Hoogerbrugge^48^ and Koelman^49^. The method consists of reducing the complexity of the atomistic description of the system through the use of a coarse-grained model. In this method, a number of atoms are combined into particles that interact with each other through soft conservative and pair wise dissipative and random forces. These forces act together as a thermostat and cause the system to rigorously sample the canonical (NVT) ensemble. The system evolves according to Newton’s equations of motion, with the force on each particle being given by the sum of the repulsive conservative force F^C^, the dissipative force F^D^, and the impulsive force *F*^*R*^:


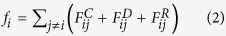


The conservative force is a soft repulsion acting along the line of centres and is given by


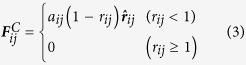


Where *a*_*ij*_ is a maximum repulsion between particle *i* and particle *j*, ***r***_*ij*_ = ***r***_*j*_ − ***r***_*i,,*_*r*_*ij*_ = |***r***_*ij*_| and ***r**ˆ*_*ij*_ = ***r***_*ij*_/|***r***_*ij*_|^23^,^45^,^50^,^51^. The inter-particle force is repulsive over a limited range only and acts in the direction of the ***r***_*ij*_ vector. The parameter *a*_*ij*_ represents the strength of the interaction. As reported previously^48^,^52^,^53^,^54^, *a*_*ij*_ can be related to the Flory Huggins interaction parameter *χ*_*ij*_:


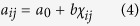


Where *a*_*0*_ = 25 and *b* = 3.27 (for a set density *ρ* = 3.0)^51^,^54^,^55^,^56^

The remaining two forces are a dissipative or drag force and a random force. They are given by


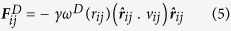


For ***r***_*ij*_ < ***r***_**c**_ and is ze***r***o otherwise. The dissipative force given in Eq. (5) also acts in the direction of ***r***_*ij*_ and is p***r***oportional to the component of the relative inter-particle velocity that lies along the ***r***_*ij*_ vector. *v*_*ij*_ = *v*_*i*_ − *v*_*j*_ is the *v*elocity difference between –particles *i* and *j*^51^. The parameter *γ* is the drag coefficient and *ω*^*D*^*(r*_*ij*_) is a “switching function” which turns off the drag force at a suitable inter-particle separation. Finally the random force is given in Eq. (6):


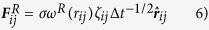


which also acts along ***r***_*ij*_, depends on a magnitude parameter *σ*, a second switching function *ω*^*R*^*(r*_*ij*_), the reciprocal square root of the simulation time step t, and a random number *ζ*_*ij*_, which has a zero average value and a unit variance. Otherwise, *ω*^*D*^ and *ω*^*R*^ are r-dependent weight functions vanishing for *r* > *r*_*c*_ = 1. These forces also act along the line of centres and conserve linear and angular momentum. There is an independent random function for each pair of particles^23^.

All forces vanish beyond the cutoff radius, *r*_*c*_. Español and Warren^57^ showed that the fluctuation–dissipation relation requires that:


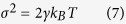


And the dissipative and random weight functions take the general form^23^,^57^,^58^:


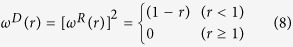


When modeling polymers or molecules, the integrity of the chain or the molecule is ensured by an additional spring force between neighboring particles given by


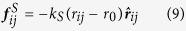


Where the equilibrium bond distance r_0_ is zero and the spring constant *k*_*s*_ = 4.0^59^,^60^. In this expression, the parameter *k*_*s*_ is associated with the strength of the string interaction and is expressed in reduced units, *k*_*B*_*T/r*_*c*_^2^. And r_0_ is the reduced equilibrium distance between particles i and j. The spring constant is chosen such that the mean distance between connected particles coincides with the point where the pair correlation function has its maximum. If we choose k_s_ much larger, the particles are tied together at very short distance and we get very stiff chains, and if we take it much smaller we get a longer distance between the connected particles than between unconnected nearest neighbors. For *ρ* = 3 and *a*_*ii*_ = 25, this occurs for *k*_*s*_ = 4. With this value, it turns out that the distance from a bead to a bonded and a non-bonded neighbor is approximately the same, which avoids having to introduce another length scale^54^. This pair wise force^61^ is then added to the sum of the DPD conservative force of Eq. (3).

**Figure 5:**
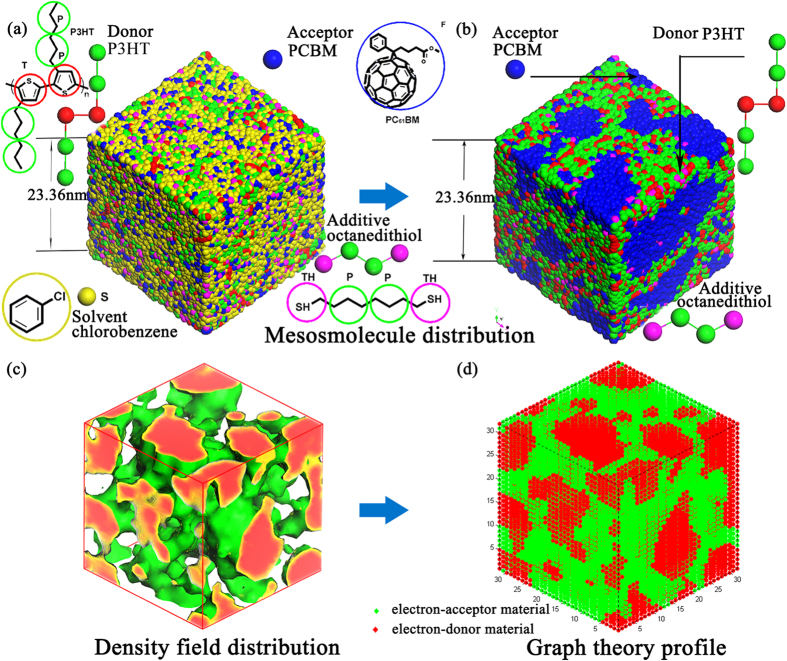
The coarse-grain models of BHJ P3HT/PCBM polymer solar cell in DPD simulations. (**a**) Coarse-grain model with the mesomolecule before DPD equilibration. (**b**) Coarse-grain model after DPD equilibration. (**c**) The 3D bi-continuous morphology of P3HT/PCBM obtained from our DPD simulation. (**d**) The converted discrete morphology from (**c**). Each particle represents roughly the same liquid volume^20^,^70^.

P3HT/PCBM BHJ solar cells are the most studied BHJ solar cells. In the current work, we focus on the study of P3HT/PCBM active layer. The molecular structures and coarse-grained particles for the simulated P3HT and PCBM are summarized in Fig. 5(a,b). Based on the chemical structure of P3HT polymer, we represent the backbone of P3HT polymer by particle T and the side chain by two connective P particles. The polymerization degree of P3HT is set as 30. Considering the Gaussian equivalent chain is used in our P3HT model, we determine the characteristic ratio of P3HT (3.49) from Synthia module of MS5.0. Thus the polymer model in our simulation represents molecular weight roughly 17.5 kg/mol. The PCBM molecule is represented by single particle F as showed in Fig. 5(a). The properties of F, P and T particles are summarized in Table S1 in Supplementary Table S1. Particle F represents bigger fragment than particle P and T. However, in DPD simulations, the size of each particle is not one of the variables affecting the calculations. Instead, the size effect is considered in the inter-particle soft potential. Thus accurate inter-particle potentials are critical for DPD simulations. We derive the particle-particle interaction potential parameters from full-atomic models and simulations.

To obtain the solubility parameters and interaction potential parameters of particles T, P, and F, we construct the amorphous structures of P3HT and PCBM and perform molecular dynamics with COMPASS force-field^62^,^63^ by using the Forcite module of Materials Studio 5.0 software package (MS5.0) to obtain cohesive energy and solubility parameters (*δ*_*i*_)^64^,^65^,^66^. The details of molecular dynamics simulations can be found in Supplementary Information S2.

The Flory-Huggins parameters (*χ*_*ij*_) between different particles are calculated based on solubility parameters (*δ*_*i*_) and the corresponding repulsion parameters (*a*_*ij*_)^64^ calculated from Eq. (4) among each particles are listed in Table 1.


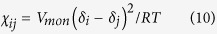


DPD calculation method is comparatively efficient; in order to get the optimized equilibrated simulation of the system efficiently, namely taking adequate simulation time steps for the certain system to get final equilibration in the minimum duration (physical time), we calculate the physical time scale of the simulation with the *r*_*c*_ of the smallest particle P. Thus, we find the physical size of the interaction radius,


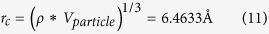


In the simulation, the particle mass, temperature, interaction range are chosen as units of mass, energy and length, hence *m* = *k*_*B*_*T* = *r*_*c*_ = 1.0^55^, m_0_ = 40 g·mol^−1^, T = 298 K, which represents the mass of the smallest real P particle in the mesoscopic model. The real length of the simulation box (*r*_*c*_) is estimated from the volume of uniformed DPD particle, where ρ* is the reduced density (a set density ρ* = 3.0) of DPD particles, *V*_*particle*_ = 90 Å^3^. The unit of time *τ* = 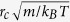 = 2.585 ps (*m* = *m*_*0*_ */N*_*A*_). The simulation time step is 0.02.

In order to investigate the effect of the DPD simulation time on our results, we perform a series of DPD simulation with different time steps and make comparison. The details can be found in Supplementary Information S3. We conclude that 20000 time steps are enough for the equilibration of the system we studied. We use 50000 as the standard simulation time steps for our DPD simulations. Because the time step is 0.02 τ which is about 50 fs, the typical simulation trajectories of 50000 time steps is roughly approximated 2.5 ns.

Here, we assume that the initial structure is homogeneous. The total number of the particles in the system is 98325; The initial simulation box has dimensions of 32 × 32 × 32 r_c_^3^ in DPD units (23.36 × 23.36 × 23.36 nm^3^), with periodic boundary conditions in all three dimensions. It will be interesting and challenging to simulate a box of 100 nm × 100 nm ×100 nm, which is about 64 times larger than our current model. In this way, we could apply electrodes above the simulation box and below the simulation box. And apply periodic boundary conditions on x, y directions, but not z direction.

### Characterization of morphology based on morphology descriptors

Wodo *et al.*^17^ presented a graph-based framework and define three meaningful physical descriptors to link the morphology descriptors and the performance of Organic solar cells (OSCs) active layer. They have explicitly proved that the “performance indicator” generated by graph theory is a good marker for the short circuit current in their model^17^. There are some references showing that the open circuit voltage (*V*_*oc*_) is related to the Donors’ HOMO and the Acceptors’ LUMO^67^. However the morphology will not affect *V*_*oc*_ directly^68^. In addition, Li *et al.*^68^ indicated that fill factor (FF) is mainly affected by charge carrier mobility and morphology of the system.

Here we use similar approach to characterize the 3D morphologies obtained from our DPD simulations.

After obtaining the 3D continuous morphology from DPD simulation as shown in Fig. 5(c), we convert the 3D continuous morphology into a discrete morphology as shown in Fig. 5(d). The basic idea is that, in each discrete unit cube, if donor shows the biggest density, we classify the whole unit cube as a donor cube. Conversely, if acceptor/solvent shows the biggest density, we classify the whole unit cube as an acceptor/solvent cube. When we estimate the performance indicator by using graph theory, we simplifies the P3HT domain into grids and don’t consider the details of the interaction between adjacent P3HT molecules.

Then we use the following four descriptors to characterize the discrete morphology.

A. Fraction of donor materials *f*_*abs*_ ---- Only the donor materials can absorb the light and generate the excited hole-electron pairs (excitons) in the active layer. So this value determines the performance indicator of generation of excitons directly.

Mathematical Representation:


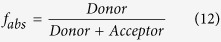


A*: The intensity light exponentially decays along when the incident photons traverses through the active-layer. So we should give an index to modify the generation of excitons as the following.


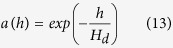


*h*: the vertical height, *H*_*d*_: the thickness of the active layer.

If there is only donor and acceptor in the morphology, we could use 0 or 1 to represent donor or acceptor, respectively. However, our study involves solvent/additive in addition to donor and acceptor. It is necessary for us to modify descriptor *f*_*abs*_ to indicate the fraction of average donor/acceptor density.


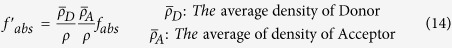


B. Fraction of donor materials whose distance to D/A interface is within a given range (particularly within the exciton diffusion length) *f*_*diss*_ ---- Because the excited hole-electron pairs has a limited lifetime or can diffuse a limited length (10 nm∼), they will recombine or become into the ground state if they cannot reach the D/A interface. So the fraction of donor material in a given range has an impact on the performance indicator of excitons dissociation.

Mathematical Representation:


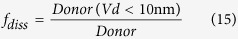


C. Fraction of materials connected to the corresponding electrodes at the interface *f*_*out*_ ---- Because when an exciton dissociates into a free hole and a free electron, it needs a continuous path to travel to the corresponding electrodes. So this fraction will affect the collection performance indicator of the electrons/holes.

Mathematic Representation:


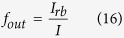


D. In addition, here we are differentiating the performance indicator of morphologies under different conditions, which involves the change of interface area and domain size in the morphology continuously. (See Supplementary Information S5) The size of the interface area between donor domain and acceptor domain is critical for the performance indicator. When the interface area is small and the domain size is big, the donor domain and the acceptor domain will be continuous and form bi-continuous pathway for charge transport. It is beneficial for “charge collection performance indicator”. When the interface area is big and the domain size is small, it will be easy for the dissociation of the exciton at the interface. It is beneficial for “exciton dissociation performance indicator.” Thus there is an optimum interface area of the morphology for the performance indicator. Young Min Nam *et al.*’s experimental results^16^ show that, when the domain size is about 6 nm, the active layer shows the optimum PCE. Meanwhile, Watkins *et al.*^69^ use dynamical Monte Carlo model to evaluate the relationship between interface area and efficiency. Watkins *et al.* evaluated the “exciton dissociation efficiency” and “charge collection efficiency” separately and they conclude that there is an optimum interface area. We use the relationship of interface area and internal quantum efficiency derived from Watkins *et al.* and the optimum domain size 6 nm based on Young Min Nam *et al.*’s experimental results. And the descriptor is defined as the following:


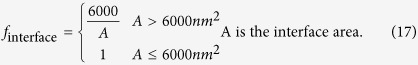


On the contrary, if we don’t include *f*_*interface*_ in the calculation, we observe a linear relationship between the interface area and the performance indicator as shown in Supplementary Information Figure S4a. Thus it will not consider the two competing factors “charge collection performance indicator” and “exciton dissociation performance indicator”. However, after we include *f*_*interface*_ in the calculation, we obtain the dependence of the performance indicator on the interface area as shown in Supplementary Information Figure S4b, which is consistent with Young Min Nam’s experimental results and Watkins’s DMC results.

In summary, the total performance indicator based on morphology descriptors is defined as:


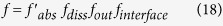


## Additional Information

**How to cite this article**: Du, C. *et al.* Morphology and Performance of Polymer Solar Cell Characterized by DPD Simulation and Graph Theory. *Sci. Rep.* **5**, 16854; doi: 10.1038/srep16854 (2015).

## Supplementary Material

Supplementary Information

## Figures and Tables

**Table 1:** The repulsion parameters (*a*_*ij*_) calculated from Flory-Huggins *χ*_*ij*_ parameters.

	PC_61_BM (F)	Propyl(P)	Solvent(S)	Thiophene(T)	CHSH(TH)
PC_61_BM(F)	25.00				
Propyl(P)	39.89	25.00			
Solvent(S)	25.22	29.61	25,00		
Thiophene(T)	25.00	29.91	25.04	25.00	
CHSH(TH)	25.21	29.85	25.20	25.06	25.00

^*^a_*ii*_ is set to 25^54^,^55^.
