# Supplementary material for: Morphology and Performance of Polymer Solar Cell Characterized by DPD Simulation and Graph Theory
Source: Sci Rep. 2015 Nov 19;5:16854. doi: 10.1038/srep16854 (PMC4652231; doi:10.1038/srep16854)
Supplement: Supplementary Information [file srep16854-s1.pdf]

Supplementary information

Morphology and Performance of Polymer Solar Cell Characterized by DPD Simulation and Graph Theory

Chunmiao Du, Yujin Ji, Junwei Xue, Tingjun Hou, Jianxin Tang, Shuit-Tong Lee, Youyong Li*,

Institute of Functional Nano and Soft Materials (FUNSOM), Soochow University, Suzhou 215123, P. R. China

Email: yyli@suda.edu.cn

S1. Graphical Abstract

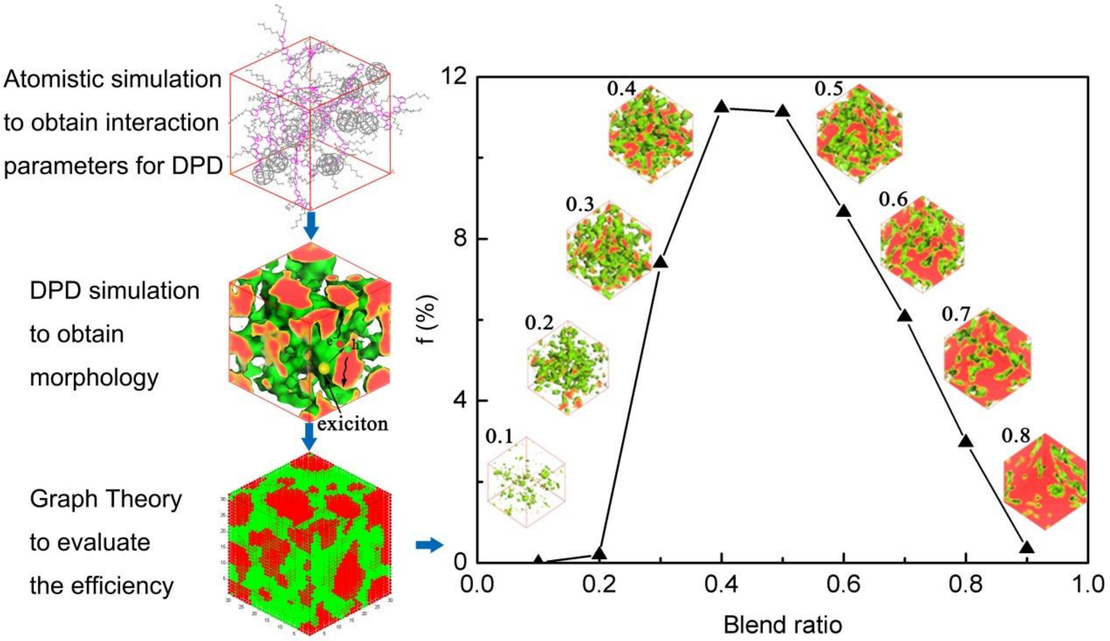

We first perform atomistic molecular dynamics simulation to obtain the interaction parameters for the components of the active layer of Organic Photovoltaics (OPV). Then we perform Dissipative Particle Dynamics (DPD) to obtain the equilibrated morphology of the active layer of OPV. Based on the predicted 3D morphology, we estimate the performance indicator by using graph theory. Here we study P3HT/ PCBM BHJ solar cells and investigates the important parameters affecting device performance such as: blend ratio, temperature, solvent, and additives. We obtain the optimum values for the parameters and domain size, which are shown to be consistent with experimental results. Our approach provides a direct method to predict dynamic morphology and performance indicator for BHJ solar cells.

S2. Molecular Dynamics Simulation of Atomistic Model

We optimize the geometric construction of each divided moiety of molecule fragment with the Forcite module in MS5.0. Then the cubic boxes of pure molecular fragments including thiophene (T), propyl (C3H), solvent (chlorobenzene), CHSH (TH) and [6,6] -phenyl C61-butyric acid methyl ester ($PC_{61}BM$) are constructed with the Amorphous package of Materials Studio 5.0 (MS5.0), followed by geometry optimization with the energy convergence threshold of 1×10^{-4} kcal·mol^{-1}. An

amorphous cell structure of PCBM is shown in Figure S1. A preliminary dynamics running of 100 picoseconds (ps) in Isothermal-Isobaric (NPT) ensemble is performed to bring the system to equilibrium stage at a constant pressure of 1 atm. Afterwards, a series of production running of 200 picoseconds (ps) in the NPT ensemble are followed, during which trajectories are stored per 1 ps for analysis. By performing molecular dynamics of atomistic model, we obtain thermodynamic parameters, including molar volume, solubility parameter, and Flory-Huggins parameter.

The interactions parameters are calculated with the COMPASS[1,2] force field. The initial velocities are generated by Maxwell distribution and the temperature and pressure are normalized by Nose and Berendsen method respectively. The Ewald summation is adopted for the Coulomb interactions with an accuracy of 0.001 kcal•mol^{-1}, and the atom-based summation is applied for the van der Waals interactions with a cut-off distance of 12.5 Å, a spline width of 1 Å, and a buffer width of 0.5 Å. For each configuration from the amorphous construction results (10 frames), MD simulation is conducted by Forcite module of MS5.0. The temperature of all MD simulations is set to 298K, with time step 1 femtosecond.

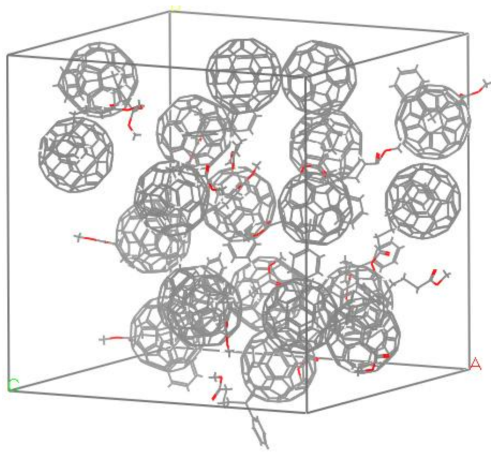

Figure S1. Simulation cell of $PC_{60}BM$ component constructed by Amorphous Builder of (MS5.0). The carbon, hydrogen and oxygen atoms are colored in gray, white and red respectively.

Based on the chemical structure of P3HT polymer, we represent the backbone of P3HT polymer by particle T and the side chain by two connective P particles. The

PCBM molecule is represented by single particle F as showed in Figure 5 in the main text. The properties of F, P and T particles are summarized in Table S1.

Table S1. The properties of F, P and T particles

	**Mass (g mol^{-1})**	**Molar volume (cm^3/mol)**	**Solubility parameter(Jcm^{-3})$^{0.5}$**	**Radius(Å)**
**PC$_{61}$BM(F)**	911	588	20.10	6.15
**Propyl(P)**	40	74	13.10	3.09
**Thiophene(T)**	80	82	16.22	3.19
**Solvent(S)**	112	102	19.40	3.43
**CHSH(TH)**	44	50	20.80	2.71

Solubility parameter[3] (δ) is a characteristic of a polymer used in predicting the solubility of that polymer in a given solvent. The solubility parameter is related to each component's volumetric cohesive energy density E_{coh}/V of the system via the definition:

$$\delta = \sqrt{E_{coh}/V} \qquad (1)$$

For a substance of low molecular weight, the value of the solubility parameter is often estimated from the enthalpy of vaporization; for a polymer, it is usually taken to be the value of the solubility parameter of the solvent producing the solution with maximum intrinsic viscosity or maximum swelling of a network of the polymer.

Molecular dynamics simulations of a series of pure systems are carried out to estimate the solubility parameters. The solubility parameters for T (thiophene), P (propyl), S (solvent), TH(CHSH), and F (PC$_{61}$BM) components are estimated to be 16.22 13.10, 19.40, 20.80 and 20.10 (J·cm^{-3})$^{0.5}$ based on our simulation results. Similarly, the Hansen solubility parameters for C$_{60}$ have been found to be 20.09 MPa$^{1/2}$[4]. Therefore, such MD simulation with COMPASS force field provides us reasonable solubility parameters.

The Flory-Huggins χ parameter could be estimated from:

$$\chi = \frac{V_{mono}}{RT}(\delta_A - \delta_B)^2 \quad (2)$$

Where V_{mono} is a monomer unit volume. Here we use the average molar volume of the two different components.

We use the relationship $a_{ij} = a_0 + b\chi_{ij}$ (a_0 = 25 and b = 3.27) to determine the corresponding repulsion parameters used in the DPD simulation and the results are summarized in Table 1 in the main text.

S3. The effect of DPD simulation time

In order to investigate the effect of the DPD simulation time on our results, we perform a series of DPD simulation with 5000, 10000, 20000, 50000, 100000 time steps and make comparison. The equilibrated morphologies with different amount of solvent after different DPD simulation time steps are summarized in Figure S2.

In Figure S2, the percentage X_S indicates the amount of the solvent in the simulated system. The simulated equilibrated morphologies with different amount of the solvent are significantly different from each other. When the simulation time steps are increased from 5000, to 10000, or 20000, 50000, 100000, the simulated equilibrated morphologies show non-negligible difference. We plot time evolution of the pressure with respect to DPD simulation time steps, as shown in Figure S3. We can see that the pressure converges after 50000 time steps.

Thus we use 50000 as the standard simulation time steps for our DPD simulations.

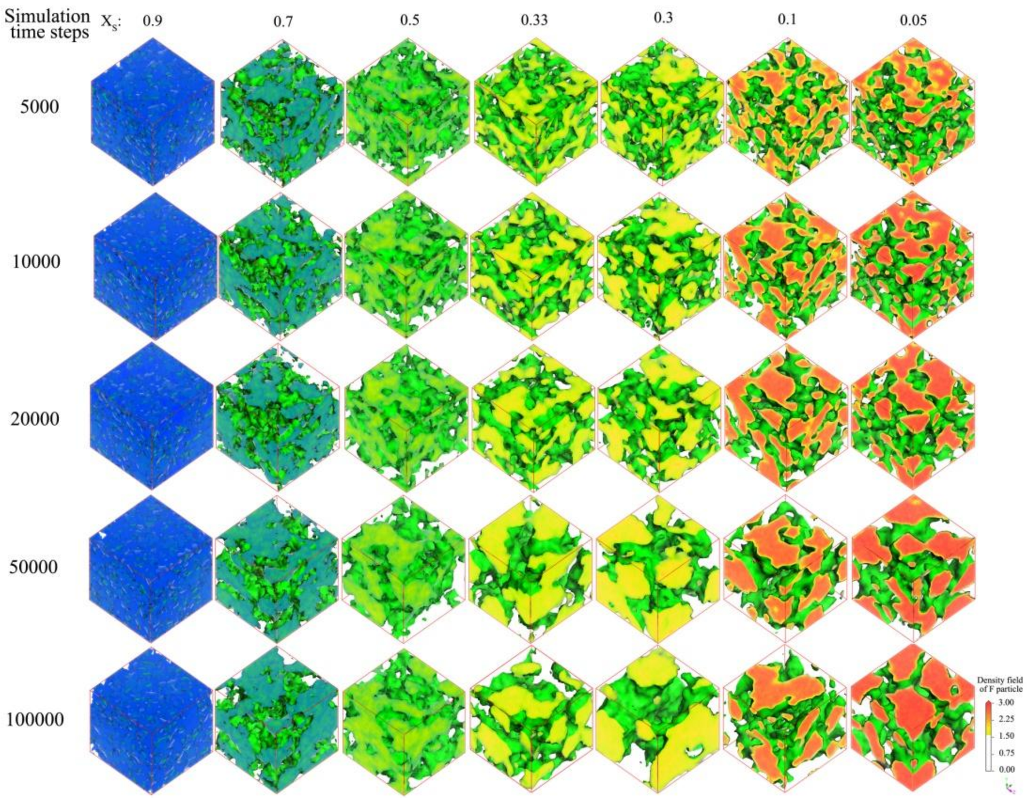

Figure S2. The equilibrated morphology with different amount of solvents (Xs) and we make comparison for the effect of the simulation time steps (5000, 10000, 20000, 50000, 100000) on the equilibrated morphology.

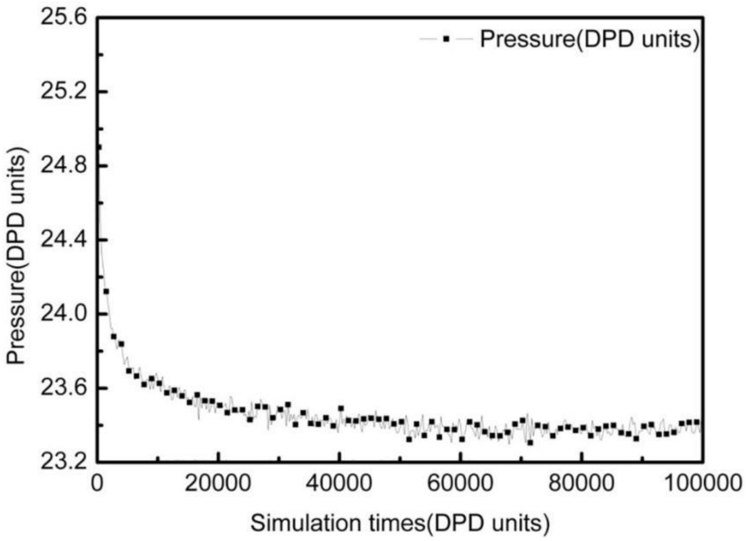

Figure S3. The evolution of the pressure with respect to DPD simulation time.

S4. The Perl script used to evaluate the order parameter.

The order parameter is defined as in the following equation:

$$P_i = \frac{1}{V}\int_V [\eta_i^2(r) - \eta_i^2]\, dV \quad (1)$$

Where η_i is a dimensionless density volume fraction for species i, $\eta_i(r) = \nu\rho_i(r)$ where $\rho_i(r)$ represents the practical local physical density for species i, ν is a scale factor between the local density $\eta_i(r)$ in DPD and $\rho_i(r)$. The order parameter defined here reflects the degree of phase separation.

```
#!/user/bin/perl
open(Input, "filename.den") or die;
open(Output, ">>G:/5wF.txt") or die;    #output file
my $valueN = 0.;
my $valueAv = 0.;
my $valueAvSqr = 0.;
my $numberLine=0;
while($line=<Input>) {
    chomp $line;
    if ($numberLine >= 1) {
            $valueN++;
            $valueAv += $line;
            $valueAvSqr += $line*$line;
    }

    $numberLine++;
}
# normalize
$valueAv /= $valueN;
$valueAvSqr /= $valueN;
# the unnormalized order parameter
my $orderParameter = $valueAvSqr-$valueAv*$valueAv;

print Output "$orderParameter \n";
close(Input);
close(Output);
```

S5. The introduction of $f_{interface}$ morphology descriptor

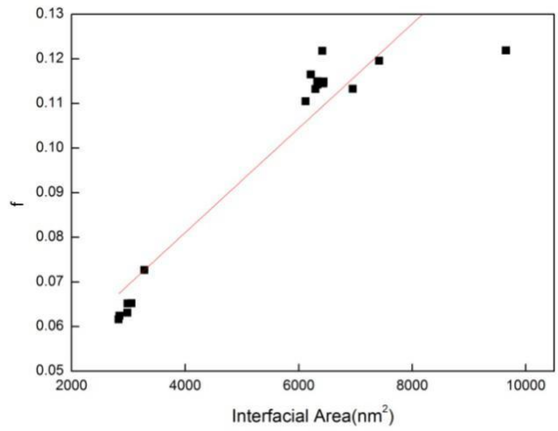

Figure S4a. The dependence of the performance indicator on the interface area, when we don't include $f_{interface}$ descriptor.

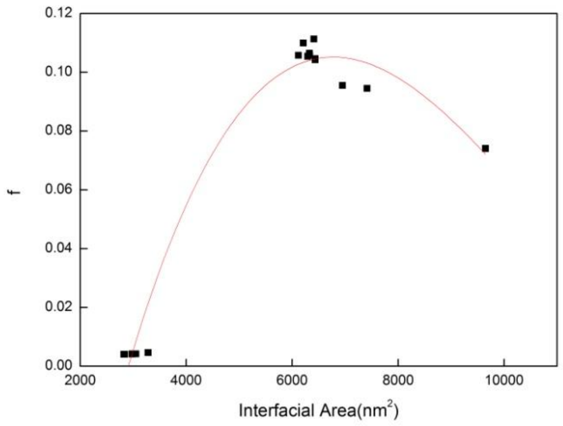

Figure S4b. The dependence of the performance indicator on the interface area, when we include $f_{interface}$ descriptor.

Here we are differentiating the performance indicator of morphologies under different conditions, which involves the change of interface area and domain size in the morphology continuously. The size of the interface area between donor domain and acceptor domain is critical for the performance indicator. When the interface area is small and the domain size is big, the donor domain and the acceptor domain will be continuous and form bi-continuous pathway for charge transport. It is beneficial for "charge collection performance indicator". When the interface area is big and the domain size is small, it will be easy for the dissociation of the exciton at the interface. It is beneficial for "exciton dissociation performance indicator." Thus there is an

optimum interface area of the morphology for the performance indicator. Young Min Nam et al.'s experimental results[5] show that, when the domain size is about 6nm, the active layer shows the optimum PCE. Meanwhile, Watkins et al.[6] use dynamical Monte Carlo model to evaluate the relationship between interface area and efficiency. Watkins et al. evaluated the "exciton dissociation efficiency" and "charge collection efficiency" separately and they conclude that there is an optimum interface area. We use the relationship of interface area and internal quantum performance indicator derived from Watkins et al. and the optimum domain size 6 nm based on Young Min Nam et al.'s experimental results. And the descriptor is defined as the following:

$$f_{interface} = \begin{cases} \frac{6000}{A} & A > 6000nm^2 \\ 1 & A \le 6000nm^2 \end{cases} \text{ A is the interface area} \quad (3)$$

On the contrary, if we don't include interface in the calculation, we observe a linear relationship between the interface area and the performance indicator as shown in Figure S4a. Thus it will not consider the two competing factors "charge collection performance indicator" and "exciton dissociation performance indicator". However, after we include interface in the calculation, we obtain the dependence of the performance indicator on the interface area as shown in Figure S4b, which is consistent with Young Min Nam's experimental results and Watkins's DMC results.

S6. The Calculation of Spatial Orientation Correlation Function (SOCF)

We calculate "Spatial Orientation Correlation Function" (SOCF) to characterize the crystallization in our simulation box. The results indicate that the degree of crystallization is low due to the small simulation box in our system.

"Orientation Correlation Function" (OCF) is defined as the correlation of two chemical bonds on the backbone of the polymer chain (either intra-molecule or inter-molecule):

$$P_2(\cos\theta) = 1/2\,(3\cos^2\theta - 1) \quad (4)$$

A perfect alignment corresponds to P=1 (angle θ is 0 or 180), whereas a random orientation gives a value of 0. In general, the larger the parameter (OCF), the more

ordered is the structure. A typical liquid crystal has an order parameter in the range 0.3 to 0.8. Lee *et al*[7] indicated that P3HT sub-domain was considered to be crystalline if the orientational order parameter is in the range 0.3 to 1.

To characterize the orientation correlation between two molecules, the order parameter is usually expressed as a function of the distance between two molecules, r:

$$S(r) = < P_2(\cos\theta) >_r = < \frac{3}{2}(\cos^2\theta - \frac{1}{3}) >_r \quad (5)$$

Where the brackets indicate an average and the subscript means that only distances of r are taken into account. The function *S(r)* is called "the spatial orientation correlation function" (SCOF). At large distances the function usually becomes zero, indicating a loss of orientation correlation as the molecules are further apart.

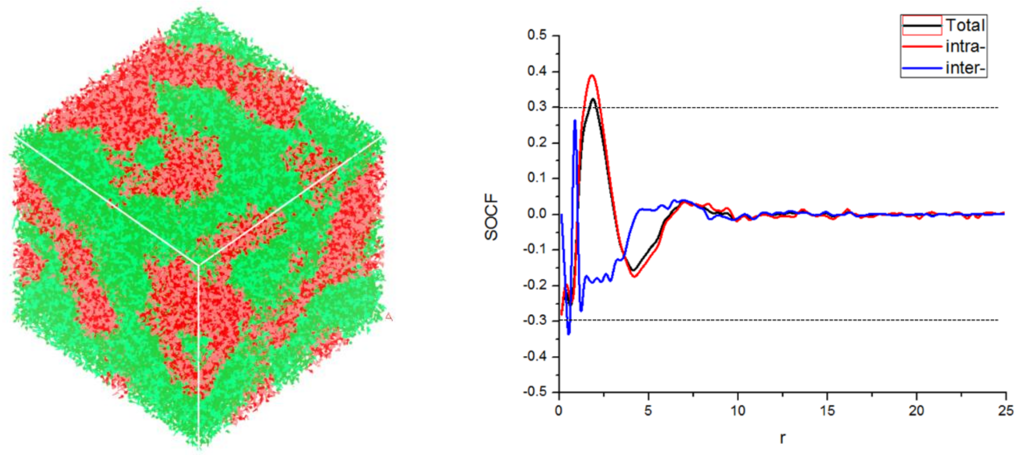

(a) (b)

Figure S5. (a) The simulation box of P3HT and PCBM without additive at the temperature 298K. (b) The SOCF changes with the distance for intra-, inter-, and total polymer chain of P3HT calculated from the simulation box shown in Figure 3a.

In Figure S5a, we show a typical simulation box of P3HT and PCBM without additive at the temperature 298K. In Figure S5b, we show the calculated SOCF as the function of distance r based on the simulation box shown in Figure S5a. At large distances the function usually becomes zero, indicating a loss of orientation correlation as the molecules are further apart. For intermediate distances, there is typically some order, in particular in the first coordination shell. In Figure S5b, for

intermediate distances, there is some order. But the calculated SOCF is still below 0.3, typically. A typical liquid crystal has SOCF in the range 0.3 to 1.[7] Thus the degree of crystallization in our simulation box is low, due to the limited size of our simulation box.

1 Sun, H. COMPASS: An ab initio force-field optimized for condensed-phase applications - Overview with details on alkane and benzene compounds. *J. Phys. Chem. B* **102**, 7338-7364 (1998).

2 Rigby, D., Sun, H. & Eichinger, B. E. Computer simulations of poly(ethylene oxide): force field, pvt diagram and cyclization behaviour. *Polym. Int.* **44**, 311-330 (1997).

3 Koenhen, D. M. & Smolders, C. A. The determination of solubility parameters of solvents and polymers by means of correlations with other physical quantities. *Journal of Applied Polymer Science* **19**, 1163-1179, doi:10.1002/app.1975.070190423 (1975).

4 Hansen, C. M. & Smith, A. L. Using Hansen solubility parameters to correlate solubility of C60 fullerene in organic solvents and in polymers. *Carbon* **42**, 1591-1597 (2004).

5 Nam, Y. M., Huh, J. & Jo, W. H. Optimization of thickness and morphology of active layer for high performance of bulk-heterojunction organic solar cells. *Sol. Energy. Mater. Sol. Cells* **94**, 1118-1124 (2010).

6 Watkins, P. K., Walker, A. B. & Verschoor, G. L. B. Dynamical Monte Carlo Modelling of Organic Solar Cells: The Dependence of Internal Quantum Efficiency on Morphology. *Nano Lett.* **5**, 1814-1818 (2005).

7 Lee, C.-K., Pao, C.-W. & Chu, C.-W. Multiscale molecular simulations of the nanoscale morphologies of P3HT:PCBM blends for bulk heterojunction organic photovoltaic cells. *Energ. Environ. Sci.* **4**, 4124-4132 (2011).
